# Location Tracking of a Radio-Wave Antenna Utilizing the Radiation Pattern Recognized by Deep Network

**DOI:** 10.3390/s26092867

**Published:** 2026-05-04

**Authors:** Yifan Zhang, William W. Clark, Bryan Tillman, Young Jae Chun, Sung Kwon Cho

**Affiliations:** 1 Department of Mechanical and Material Engineering, University of Pittsburgh, 3700 O’Hara Street, Pittsburgh, PA 15261, USA; wclark@pitt.edu (W.W.C.); yjchun@pitt.edu (Y.J.C.); skcho@pitt.edu (S.K.C.); 2 The Ohio State University Wexner Medical Center, 895 Yard Street, Grandview Heights, OH 43212, USA; bryan.tillman@osumc.edu

**Keywords:** radio frequency, location tracking, antenna, pattern recognition

## Abstract

This paper will introduce a radio frequency system to track the location of a stent designed to work inside a human artery. The stent is designed as a hemostasis aid tool for emergency situations where common surgical equipment, such as fluoroscopy systems, is not available, such as on the battlefield. In the application of interest, the stent must be guided to the correct location to achieve effective hemostasis and prevent complications. The locating approach uses the radiation pattern from the transmitter as the reference. When the transmitting frequency changes over a certain range, the measurement amplitude from a receiver depends on its relative location with respect to the transmitter. However, when the input frequency is unequal to the resonance frequency, the radiation pattern varies in an unpredictable way. To solve this problem, a deep learning model was trained to recognize variations in the radiation pattern and predict the receiver’s location as one of the classes in the reference grid. The deep learning model also reduces the impact of noise and disturbing signals, which effectively improves the system’s robustness.

## 1. Introduction

In common situations where injuries occur on the body surface, the accompanying bleeding can be effectively stopped by pressing the wound area with a bandage. However, for serious wounds that occur deep within the body, such as trauma caused by a bullet or shrapnel on the battlefield, external treatments can hardly stop bleeding. Without immediate treatment, the hemorrhage could cause death before the patient reaches the hospital.

To save the lives of patients in such cases, a retrievable rescue perfusion stent was developed to swiftly achieve hemostasis. The cylindrical stent is covered with an impermeable (PTFE) layer. When deployed into the aorta, the stent expands to support the PTFE layer to cover the wound in the vessel, thereby creating an effective second interior artery wall, which has been shown to reduce 93% of hemorrhage within 10 s while blood can still flow within [1,2]. The stent was designed to be long enough to cover a large portion of the aorta to be most effective. However, such a design can cause side effects since branch vessels along the artery may be occluded by the stent. For example, when the wounded major artery is the celiac trunk, which contains branches of the hepatic, splenic, and left gastric arteries, blocking of these branches for a long time could result in fatal complications [3].

**Figure 1:**
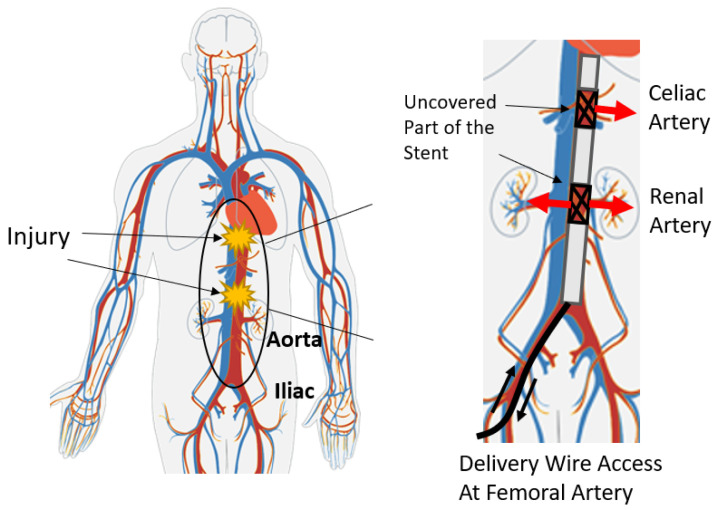
The diagram of the impermeable stent that achieves hemostasis by covering the majority of the aorta with the blood-proof layer. The location measurement of the stent is important to align the uncovered outlet with branch arteries along the aorta [4].

To avoid occlusion of the branch arteries, outlets were created in the PTFE layer, through which blood can perfuse to side branches. In practice, deployment of the stent starts by inserting it into the body through an incision in the iliac artery and then pushing it cranially into the aorta with a guide wire. As the stent is advanced, the uncovered portion must be matched to the branch arteries to keep the routes open. The celiac trunk generally lies within 1–3 cm of the xiphoid bone, which can be felt externally on the body. Therefore, the xiphoid bone is regarded as the landmark for the target location. Accurate measurement of the location of the stent with respect to this landmark is a critical requirement for this system. The diagram of this system is presented in Figure 1.

## 2. Related Work

Currently, several techniques are applied in surgical applications to track the location of the stent. For example, imaging systems such as fluoroscopy, magnetic resonance imaging, and ultrasound are widely used in hospitals. However, an obvious drawback of these systems is their inconvenience due to their large size, especially for emergent situations like on the battlefield. Alternatively, many portable systems utilize an electromagnetic field as the measurement medium. Some applications apply a permanent magnet as the measurement reference. For example, ref. [5] applied a quasi-static magnet as the reference. The sensor location is obtained by searching the real-time measurements in the lookup reference to find the closest matching point. The system also includes inertial sensors to measure the orientation of the stent. Alternate applications of magnetic field have used a high-frequency electromagnetic field (EMF) as the reference signal, which induces a current in the sensing coil near the EMF source. A drawback to these approaches is that measurement with the magnetic field is degraded when the reference magnetic field is distorted, which is common for both types of reference magnetic field when used in the presence of electrical machinery [6].

According to the Biot–Savart law, regardless of the reference EMF frequency, the measurement from the sensor can be expressed as a function that takes its relative position with respect to the EMF sources as parameters. The parameters usually include three location coordinates, three orientation angles, and application-specific noise terms. Solving for them requires a greater number of equations than the number of parameters. Therefore, the EMF source usually includes multiple coils bound concentrically with a certain angular offset. Then the solution can be obtained by applying the least-squares algorithm, which assumes constant error across multiple measurements, thus limiting the correction frequency for strong or fast-moving disturbances. It is a common strategy to increase the number of EMF sources and sensors to improve the measurement resolution and dimension. For example, ref. [7] applied a system with 3 EMF sources and 25 sensors to measure the 5-D motion. Instead of the absolute EMF strength, ref. [8] applied the gradient of EMF in space as a reference for location measurement. Regardless of the principle, these systems work in the near-source range, where the EMF magnitude decays proportional to $1/r^{3}$ (*r* is the transmitter-receiver distance) [9]. To solve these problems, we developed a system in which the EMF source operates in the radio frequency (RF) spectrum (20 kHz to 300 GHz). This spectrum solves the problem for commercial trackers that work below 20 kHz, while maintaining the signal-penetration ability in surgical applications. This spectrum is used for location measurement in radio frequency identification (RFID) systems, where tags are placed at desired locations. This system is very strong against disturbances, while the resolution is usually low due to the sensor’s physical size [10]. The extended EMF spectrum causes continuous change of the radiation pattern around the transmitting antenna. The correspondingly changed measurement pattern from the sensor is then used to determine its location.

For all applications that use EMF, keeping the reference EMF free of disturbances is always critical for maintaining measurement accuracy. The source of distortion can be any ferrous or reactive material around the EMF system. The high randomness and variation of the EMF disturbance are always the main cause of error in these systems. Especially for this application, when the source EMF frequency does not match the resonant frequency, which is determined by the physical antenna length and the transmitting wavelength, the impact of the disturbance is difficult to model and exclude [11].

Based on the current state of EMF trackers (Table 1), this study proposes a new location detection system that works in the RF spectrum. The intellectual merit comes from the following aspects that will be introduced in the following sections:

- The usage of EMF variation in a certain spectrum as the reference fingerprint for location measurement, which is more robust to disturbance signal with narrow bandwidth.
- The corresponding antenna and circuit design to maximize the fingerprint EMF variation.
- Application of a signal denoising algorithm with a deep learning (DL) model, which is capable of removing the noise without the ground truth signal as a reference.

**Table 1:** Information of common location trackers and their features.

Source Signal	Frequency	Detection Range	Limitation
Permanent magnet field strength	0	<1 m	Field strength decreases by distance
Quasi-static EMF induction	kHz	1–2 m	Disturbance from reflective metals
RFID transmitted data	kHz to MHz	Meters	Low locating resolution

## 3. Methods

### 3.1. Modeling of the EMF Pattern

**Figure 2:**
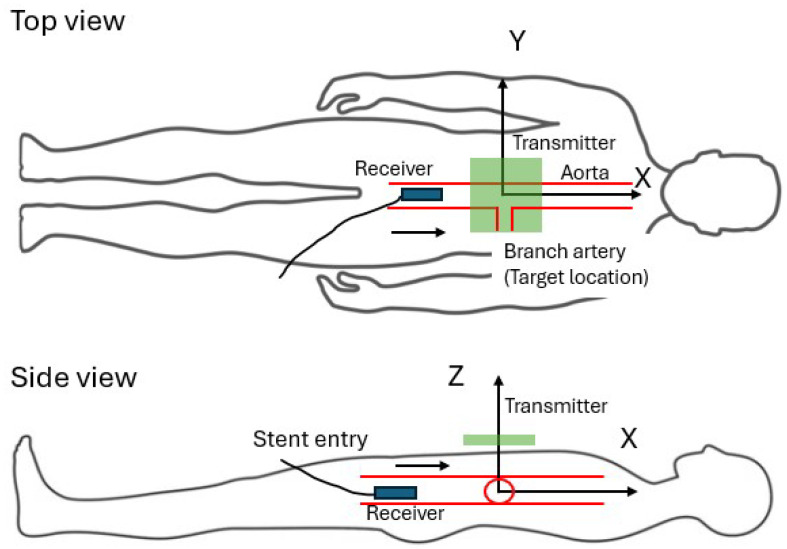
The diagram of the locating system in practice. The stent is introduced into the aorta through a lower body incision, then pushed forward into the aorta (the arrow pointing direction). The RF transmitter is placed above the target location.

EMF tracking systems usually include two essential parts: an active transmitter that radiates an electromagnetic wave and a passive receiver that catches the induced electromagnetic field. In the ideal situation, the design of the transmitter and receiver can be exchanged reciprocally. For the purpose of this application, we designed a small receiver that can fit within the diameter of the artery. The receiver is attached to the stent and is pushed forward with a guide wire from the outside of the body. The transmitter is placed at a fixed location outside the body, where there is no restriction on antenna shape and size. This design allowed for convenient adjustment of the antenna shape to generate an ideal radiation pattern. The reduced movement of the transmission cord also reduced interference. To measure the location of the receiver with respect to the target location, we defined an orthogonal coordinate frame fixed on the body of the patient. The diagram of this system is shown in Figure 2. The origin is fixed at the target location, which is the xiphoid process that can be felt by the medical operator. According to anatomy, the moving trajectory within the aorta is nearly a straight line, which is aligned with the *X* axis. The *Y* axis is perpendicular to the *X* axis in the horizontal plane, and thereby the *Z* axis points in the vertical direction. Advancement of the stent causes a primary change along the *X* direction and a trivial change along the *Y* and *Z* directions. When the transmitter is placed right above the target location (the xiphoid bone) defined as the origin, the locating objective is equivalent to determining when the receiver is at the location of X = 0.

To solve for the location of the receiver in the EMF generated by the transmitter, the knowledge of the EMF pattern in the peripheral of the transmitting coil is required. The theoretical basis is known as the Biot–Savart Law, which spatially relates the magnetic field to the current source. At one location *X*, the magnetic field generated from an infinitesimal conductor element $d\vec{L}$ is expressed by Equation (1). In this equation, $\mu_{0}$ is the magnetic permeability of free space. The current amplitude is *I*. The distance between $d\vec{L}$ and *X* is *r*, with ${\vec{l}}_{r}$ being a unit vector pointing from $d\vec{L}$ to *X* [12]. When the current amplitude is static, the radiation pattern for a transmitting coil in any shape can be obtained by integrating the infinitesimal elements along the antenna path. This analytical way of modeling the EMF is feasible when the antenna shape is simple. For example, Shao [13] simplified small circular coils as dipoles, then derived the location with the inverse equation.

(1) $$d\vec{B}=\frac{\mu_{0}Id\vec{L}\times {\vec{l}}_{r}}{4\pi r^{2}}\;$$

**Figure 3:**
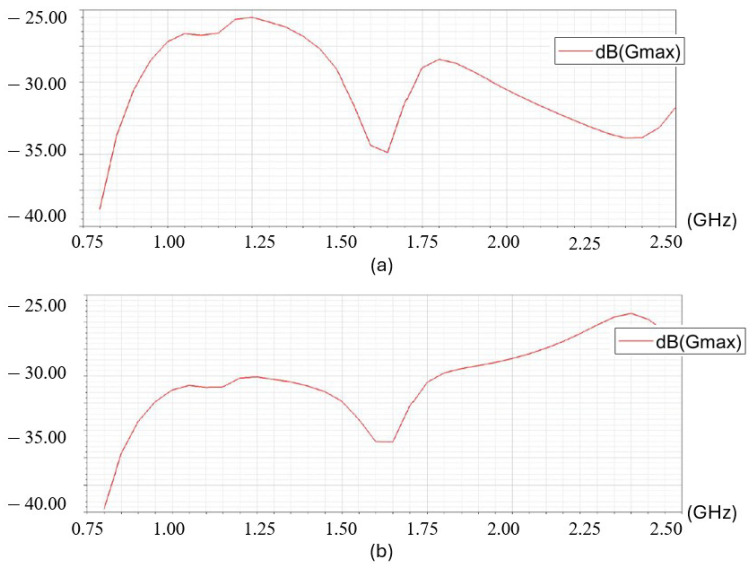
The RF spectrum around one fixed transmitter, from one receiver at two locations. (**a**) Receiver at X=−5,Y=0,Z=10 cm, (**b**) Receiver at X=+5,Y=0,Z=10 cm.

Though the EMF pattern can be modeled with an antenna in any structure using the analytical equation, many applications make reasonable simplifications regarding the actual antenna structure when they are too complicated or inaccessible. To improve the efficiency in modeling antennas with different shapes, we applied ANSYS HFSS 2020 to visualize and record the EMF pattern in space. The locating strategy is similar to [5] where the magnetic field model is paired with location coordinates. For example, for an arbitrary location where the coordinates are (x,y,z), there is a unique magnetic field vector $\vec{B}=\left[B_{X},B_{Y},B_{Z}\right]$ paired with it. All pairs of locations and $\vec{B}$ are recorded in advance and used as a lookup table to search for new measurements. For an antenna designed to a certain resonant frequency, the power transmission efficiency varies asymmetrically across the spectrum centered on that frequency. Therefore, we hypothesized that the series of measured RF power at all input AC frequencies is unique at different locations with respect to the transmitter. For example, Figure 3 shows the simulated RF power of a receiver at two different locations with respect to the transmitter (X=−5,Y=0,Z=10 cm and X=+5,Y=0,Z=10 cm). The curves are formed in the spectrum of 0.75–2.50 GHz, incremented by 0.05 GHz, which can be considered as a sequential vector $\vec{P}=\left[P_{f=0.75},P_{f=0.80},...,P_{f=2.50}\right]$. The vectors of the RF power paired with the location coordinates will be recorded in advance. Then a new measurement ${\vec{p}}_{new}$ will be compared with all references to find the closest match, thereby giving the paired location as a measurement.

### 3.2. Design of the Transmitter

Given the locating principle, the most important factor to consider in the transmitter and receiver designs is to maximize the variation of $\vec{P}$ across the spectrum with respect to the receiver location. A larger variation in the spectrum makes it easier to recognize it as a unique pattern, thus increasing the resolution and robustness of location measurement. In this research, we primarily adjusted the antenna’s shape to manipulate the radiation pattern around it. In addition, we wanted a wide bandwidth around the resonant frequency to maintain relatively high transmission efficiency, which further increases the signal-to-noise (SNR) ratio. Many types of antennas are named by their shapes, such as dipole antenna, loop antenna, T antenna, Yagi antenna, etc. In this research, we chose a loop antenna for both transmitter and receiver and modified their structures to meet these requirements.

When designing the transmitter, we first chose the system resonant frequency, at which the power is radiated with the highest efficiency [14]. We used a function generator (DSG3030, Rigol, Beijing, China) that can provide AC with frequency ranging from 9 kHz to 3 GHz, and amplitude ranging from −130 dBm to +13 dBm as the power source. While the choice of the resonant frequency can be flexible, we set the resonant frequency at $f_{r}=9$ MHz because the lower $f_{r}$ shows less attenuation and path loss under indoor conditions [15].

Next, we considered the antenna area on the basis of practical restrictions. In adult patients, the vertical distance between the transmitter and the receiver (the distance between the xiphoid bone and the aorta along the Z-axis) is commonly in the range of 10–15 cm. Here, we define the straight distance between an arbitrary field location and the transmitter as *R*, and the diagonal length of the transmitter antenna as *D*. There are two significant boundaries at the periphery of the transmitter, defined by Equations (2) and (3). The region where $R<R_{1}$ is known as the reactive near-field region, the region where $R_{1}<R<R_{2}$ is known as the radiating near-field region, and the region where $R_{2}<R$ is known as the far-field region [16]. The radiation lobes begin to form in the radiating near-field region and become well-formed in the far-field region. Therefore, it is ideal to keep measurement collection in the far-field region. Considering the requirement for a larger variation in $\vec{P}$, the size of the transmitter antenna should be designed to make $R_{2}$ greater than 10 cm. For example, as we set the resonant frequency $f_{r}=9$ MHz, the minimum size is D=12.8 cm (the wavelength λ is obtained by $\lambda =\frac{c}{f}$, where *c* is the speed of light constant).

(2) $$R_{1}=0.62\sqrt{D^{3}/\lambda }$$

(3) $$R_{2}=2D^{2}/\lambda$$

**Figure 4:**
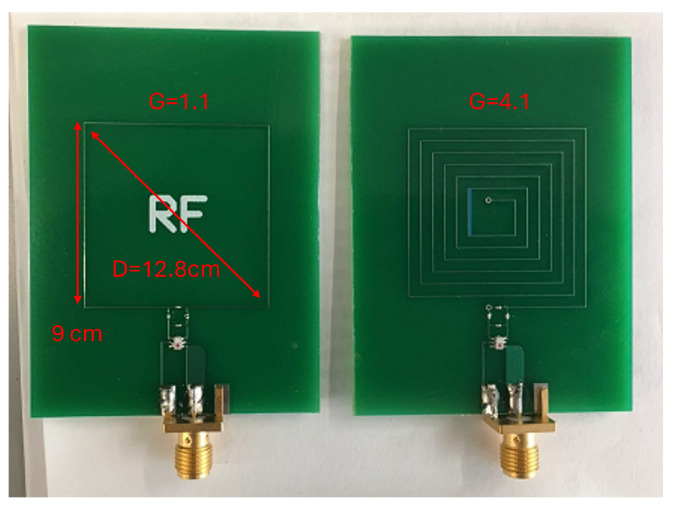
Two RF transmitters with identical area fabricated on PCBs. (**Left**): transmitter 1 with G=1.05. (**Right**): transmitter 2 with G=4.05.

It is common practice to define the electrical length *G* of an antenna as the ratio of its total conductor length *l* over the wavelength λ. *G* determines when the phase shift along the conductor becomes an important factor for the radiation pattern. When *G* is much less than 1, the antenna circuit can be regarded as a lumped element because the phase shift of AC transmission on the conductor is negligible. Otherwise, the conductor will have significant reactance relative to *G*, and a distributed-element model should be applied. According to antenna theory, when the total length of the antenna loop is close to one or more whole wavelengths at the operating frequency, the loop is regarded as a ‘large loop’ and the most efficient of all antenna types for both transmission and reception [14]. In addition, the simulation shows that a large *G* results in more side lobes in the radiation pattern and a wider bandwidth, which are beneficial in this application by providing more spatial features. However, it also makes the antenna more sensitive to minor structural variations or external disturbances, which reduces the robustness of the system against noise. To balance these two factors, we fabricated two transmitters with different antenna shapes on the printed circuit board (PCB). The first transmitter in Figure 4 has G=1.05, and the second transmitter has G=4.05. The *G* value was chosen to be slightly larger than the integer multiples of the wavelength to compensate for potential errors in manufacturing and operation processes [11].

**Figure 5:**
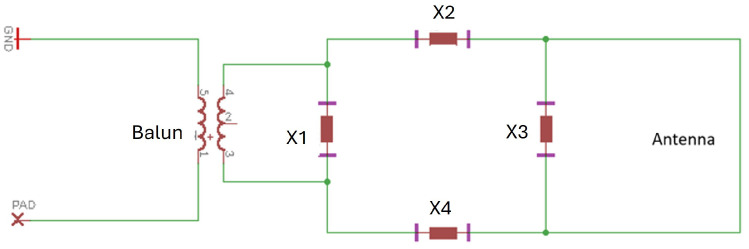
The transmitter circuit schematic diagram. $X_{1},X_{2},X_{3},X_{4}$ are the solder positions for the matching network elements.

One common problem in the RF system is interference in the background. Signals from other radiation sources, such as broadcast radio, radar systems, and power lines, can be detected by the antenna, causing disturbance to the desired signal. The interference problem can be effectively solved by balanced transmitter and receiver circuits. In balanced transmission lines, current flows with the same amplitude but opposite directions on both input and output conductors, which cancels out external interference that equally affects both conductors [17]. In our system, the transmitter is connected to the function generator through a coaxial cable, which contains four layers (from the outside to the inside): plastic jacket, metallic shield, dielectric insulator, and central core. It is called an unbalanced transmission line because the signal is transmitted to the central core, while the metallic shield is connected to the ground reference. This structure protects the signal from external interference in the metallic shield-covered range. However, the RF system still has long ranges that are not covered, especially at the antenna. Therefore, we placed a balun (balanced-to-unbalanced transformer) at the interface between the unbalanced cable and the balanced antenna, as shown in Figure 5. We chose the balun (0845BL05A0100) whose mechanical size is 0.65 mm × 0.5 mm × 0.45 mm and frequency spectrum is 729–960 MHz.

**Figure 6:**
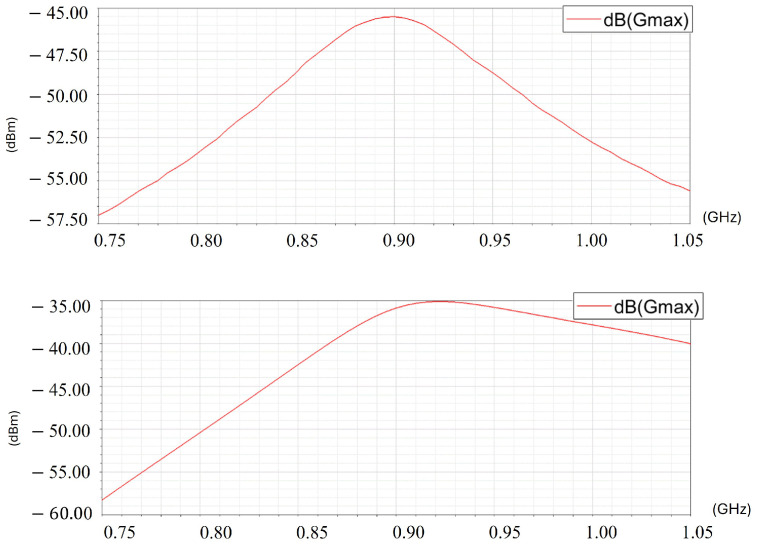
The received power amplitude. (**Top**): without impedance matching. (**Bottom**): with impedance matching.

The structure next to the balun is the impedance-matching network, which is used to match the source impedance with the load impedance. When the source impedance complex ($Z_{s}=R_{s}+jX_{s}$, where $R_{s}$ is resistance, and $X_{s}$ is reactance) conjugates with the load impedance, the transmitter reaches the highest power transfer efficiency. When the source-load impedance is not matched, part of the forward signal is reflected back and causes a standing wave along the transmission line [18]. In this system, the source impedance from the standard function generator and the coaxial cable is 50 Ω, which is the target impedance of the transmitter circuit. To reach it, we placed elements $X_{1}$ to $X_{4}$ next to the antenna in a symmetric structure to maintain circuit balance. In this research, we first used the model in ANSYS HFSS to measure the antenna impedance at any frequency. For example, transmitter 1 in Figure 4 has resistance $R_{L}=1.0\;\Omega$ and reactance $X_{L}=1.25$ Ω at $f_{r}=9$ MHz. The values of $X_{1}$ to $X_{4}$ are calculated using the approach introduced in [19], resulting in $X_{1}$ being a 10 pF capacitor, $X_{3}$ being a 3 *pF* capacitor, $X_{2}$ and $X_{4}$ being 9 nH inductors. As a result, the impedance matching network effectively increased the power amplitude within the entire range of the source AC frequency (Figure 6). With properly matched impedance, the maximum power amplitude increased from −45.5 dBm to −35 dBm at the resonant frequency. The amplitude across the entire spectrum is also increased, especially on the right-hand side of the resonant frequency. This feature makes it possible to expand the frequency spectrum to generate more features in the sequential measurement $\vec{P}$.

### 3.3. Design of the Receiver

The receiver design procedures are similar to those of the transmitter. Their circuit schematics are identical (Figure 5). Elements to be placed on $X_{1}$ to $X_{4}$ are calculated by using the impedance matching procedures introduced above. The main difference between them is the antenna electrical length. While the *G* values for the transmitters are greater than 1 to generate a characteristic radiation pattern, *G* for a receiver is typically less than 0.3 [14], which means the side length of the square loop antenna is less than 3 mm. The small size of the receiver is necessary for this application, as it will be deployed in a human artery, where the diameter is around 3 mm. In addition, the small antenna has exceptionally precise directions in which the signal vanishes (due to the parallel magnetic field and the antenna plane), which gives it great capability in direction finding [14]. The receiver is connected through a coaxial cable to an RF power detector (LTC5582IDD), which linearly converts the decibel-scaled AC power (unit dBm) into an analog DC voltage (unit V).

**Figure 7:**
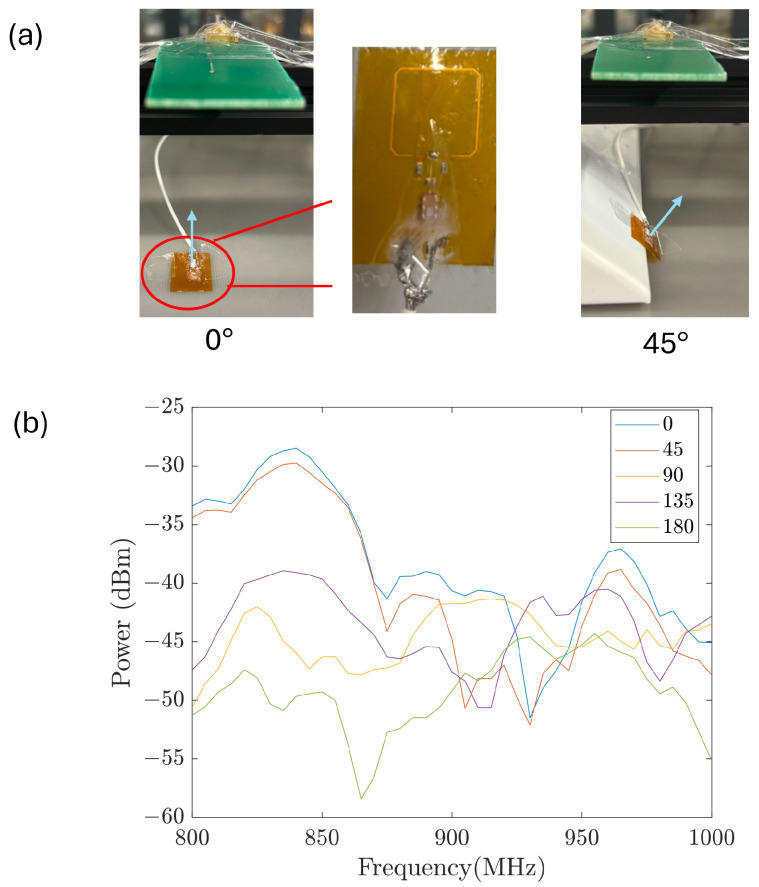
(**a**) Experiments with the transmitter-receiver angles at 0 and 45 degrees. The normal vector on the receiver shows the rotation angle. The transmitter lies horizontally above the receiver. (**b**) Power measurement with different receiver orientations.

**Figure 8:**
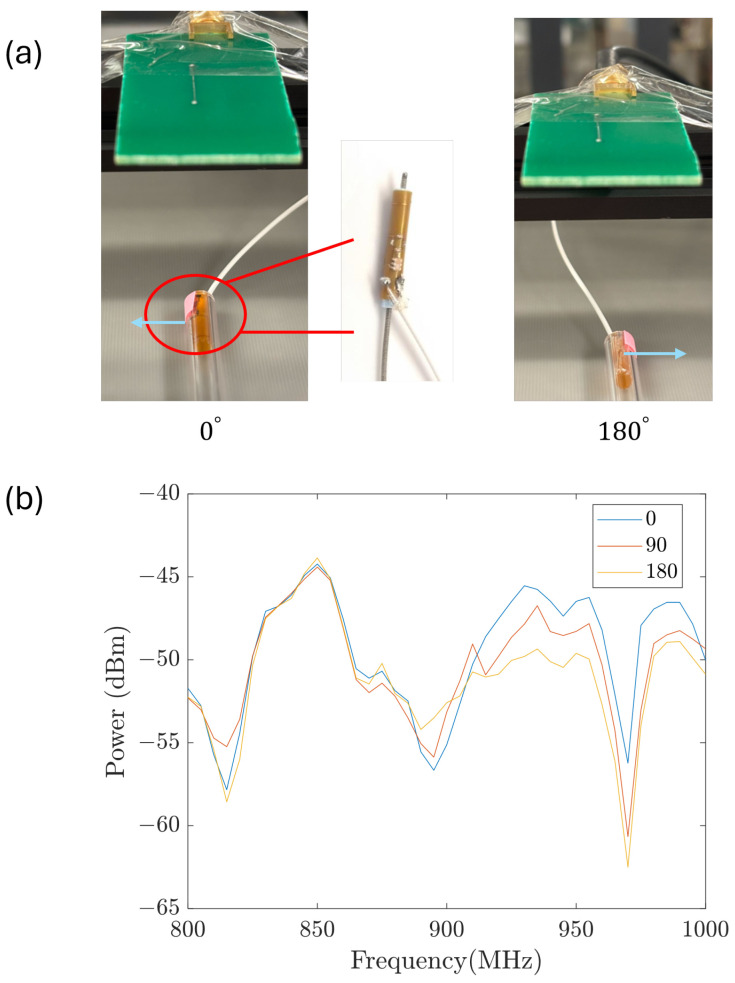
(**a**) Diagram of the transmitter-receiver angle with the receiver rolled into a cylinder. (**b**) Power measurement with different receiver orientations.

The second difference between the transmitting and receiving circuits is the substrate material. The transmitter is built on a hard PCB substrate since it is kept outside the body, while the receiver is built on a flexible, bio-compatible PCB that allows it to be bent and fit into the collecting catheter. According to Faraday’s law, the induced voltage amplitude is proportional to the antenna area perpendicular to the magnetic field vector $\vec{B}$. Rolling the antenna from a flat plane into a cylinder leaves the measurements unchanged when rotation occurs along the axial direction. This is beneficial in our application because rotation around the axial direction is hard to restrict as the stent is moved forward by pushing the guiding wire. Figure 7 shows measurements when the receiver antenna was kept flat. The axial orientation change of the receiver antenna with respect to the orientation of the transmitter antenna causes obvious differences in the measured spectra (that is, the spectra vary dramatically when the inclination angle changes between the two antennas). On the other hand, Figure 8 shows results of a similar test when the receiver antenna is rolled to form a cylinder (as is done when it is placed onto the catheter, with the cylindrical axis along the guide wire). The signal variation with axial rotation is significantly reduced. Therefore, in this study, we applied the receiver rolled around the catheter. It is worth noting that a flat antenna could be used for orientation detection, if needed, in future studies.

### 3.4. Location Measurement with Deep Learning

**Figure 9:**
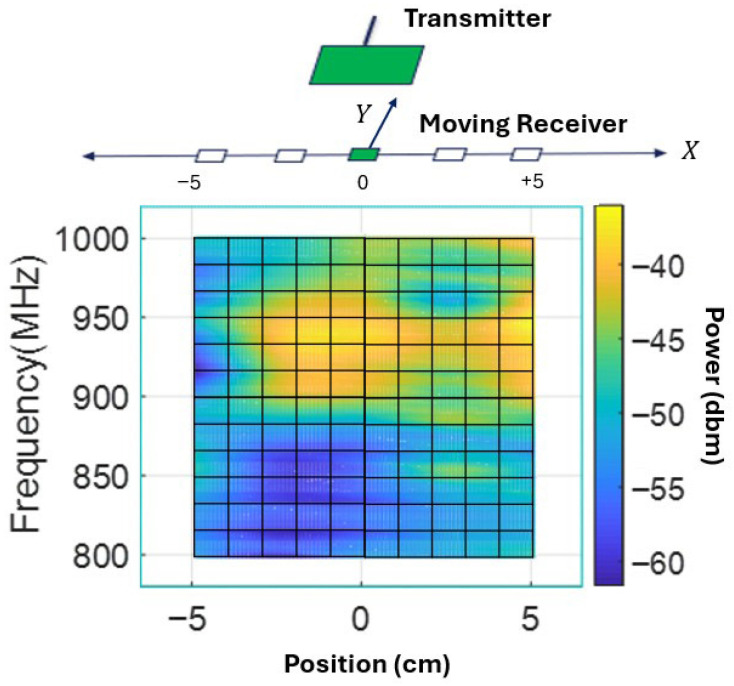
The EMF measurement along the receiver’s movement trajectory.

This section will introduce the location measurement method with the signal measured by the receiver. The idea behind this method is that the variation of the EMF in the spectrum should have a unique pattern at different field locations. A DL model is applied to recognize this pattern and match it with previously learned location labels. For example, Figure 3 showed the spectra at two different locations, which are then recognized and classified as X=−5 and X=+5. We simulated the actual motion of the receiver inside the aorta as shown in Figure 2. We simplified the actual trajectory as a linear one along the *X* axis, which is reasonable according to the primary needs of the medical technician. The receiver was moved along *X* from −5 cm to +5 cm with 1 cm increments (11 locations in total), while the transmitter was fixed 10 cm above the plane (Z=10 cm) and the location in *Y* was kept at 0. The receiver measurement spectra at all locations are shown in Figure 9. For the DL model, the target labels include the 11 different location targets that increase from X=−5 to X=+5. At each location, the input AC spectrum ranged from 800 MHz to 1000 MHz. In this way, each column in Figure 9 is matched with one location coordinate. Our goal is to use a pattern such as that shown in Figure 9 as a lookup table to determine the receiver’s location.

Ideally, with the EMF modeling approaches using either analytical equations or ANSYS HFSS, we could record and recognize the radiation pattern for antennas with arbitrary shapes and spectra, as shown in Figure 9. However, this only works when the actual measurements are disturbance-free, so that they perfectly match the reference model. In practice, even with interference-prevention strategies in place, noise can be introduced in many areas and is difficult to completely remove. First, perfect impedance matching can occur only at the resonant frequency. Working in the off-resonant spectrum attenuates the signal transmission efficiency and thereby results in a decrease in SNR. As a result, even a uniform electromagnetic disturbance would have a different impact at all frequencies. Second, the small size of the antenna in this application (especially the receiver) has a very small impedance. As a result, the soldering joints on the PCB could introduce unpredictable errors when measuring the load impedance. Third, due to the low amplitude of the transmitted signal, leakage along the transmission line, especially around unshielded joints, could become a source of interference. The leakage amplitude can be quantified in a condition in which the receiver and transmitter are stationary, and the transmission line of the transmitter is moved arbitrarily. When the transmitter works at its resonant frequency, the variation of the RF power measurement is due to the motion of the transmission line, whose amplitude could reach 20% of the reference signal. For the reasons given here, in this study, a priori experimental measurements are used as the reference for determining the location of the receiver.

In this research, we define the receiver’s measurement by Equation (4), where *f* is the frequency and *x* is the location to be measured. *g* is the modeling function to generate the reference EMF. The noise *W* is added to represent the potential disturbances introduced above. We assumed *W* follows different distributions for different types of interference, which will be introduced in the next section.

(4) P=g(f,x)+W

**Figure 10:**
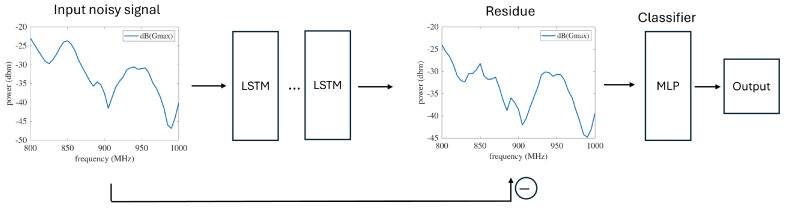
The structure of the DL network. The first part learns the residue in the noisy input signal, and the second part classifies the denoised signal to a location category.

Due to the high complexity and unpredictability of the interference, it is difficult to quantify the values of *W* and remove them. Therefore, we decided to apply a DL network that is capable of denoising even without knowing the underlying system. For example, ref. [20] introduced Noise2Self, which can denoise images without clean data and knowledge of the noise distribution. It assumes that the noise is statistically independent while the actual data are correlated across the features. This blind denoise method is very suitable in this application. For the neural network implementation, we used sequential measurements of the receiver $\vec{P}$ as input and the actual location *x* as the target belonging to a categorical series. For example, when the AC frequency changes incrementally from 800 MHz to 1000 MHz by 5 MHz, and the training data sets were collected at x=−5,−2.5,0,+2.5 and +5 cm, the dimension of the input features is 41 and the number of target classes is 5. In this research, we applied a neural network with the MATLAB (R2023b) Deep Learning toolbox without modifying the learning algorithm. The DL structure is shown in Figure 10. The network consists of two parts. The first part applies the idea of the Noise2Self framework by using the noisy signal as the input for self-supervised learning. The input is masked randomly for each batch, then followed by stacked long short-term memory (LSTM) layers (*n* = 3), which treats the spectrum as a sequential series. The first part ends with learning the residue, which is a ‘skip-connection’ between the noisy measurements (the input) and the actual signal. The loss in the first part ($L_{noise}$) is the square root of the residue of the masked samples. The second part feeds the denoised signal to an MLP classifier to generate the output. The loss in the second part is the classification error $L_{class}$. The total loss consists of these two parts weighted by $\lambda_{1}$ and $\lambda_{2}$ (Equation (5)). Since the denoise part is more essential and complex than the classification part, we assigned $\lambda_{1}=0.8$ and $\lambda_{2}=0.2$.

(5) $$L=\lambda_{1}L_{noise}+\lambda_{2}L_{class}$$

## 4. Experiment

### 4.1. The Performance of the Antenna

**Figure 11:**
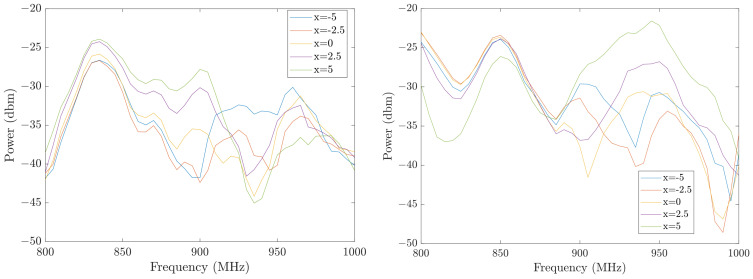
The measurements at all locations from the one-loop transmitter (**left**) and the multi-loop transmitter (**right**).

This section will validate the hardware designs and the locating algorithm with the actual system. First, we tested the two transmitter structures shown in Figure 4. As mentioned previously, increasing the electric length of the antenna could increase the heterogeneity of the EMF and increase the bandwidth, while decreasing its robustness against disturbances. Heterogeneity is quantified as the root mean square deviation (RMSD) (Equation (6)) in the same spectrum between adjacent locations. For example, in the EMF with 5 reference locations, we calculated 4 RMSDs where ${\vec{z}}_{1}$ and ${\vec{z}}_{2}$ in Equation (6) are measured at adjacent pairs x=−5 and −2.5, x=−2.5 and 0, x=0 and 2.5, x=2.5 and 5. Then, all the RMSDs are averaged as the final metric. A higher value of this metric suggests that the radiation pattern has higher variation spatially and thereby is feasible for higher locating resolution than a spectrum with lower heterogeneity. Figure 11 shows the measurements from the five reference locations using the one-loop antenna and the multi-loop antenna transmitters. The averaged RMSD between adjacent locations for the two antennas is 2.61 and 3.62, respectively, which suggests the radiation pattern from the multi-loop antenna has a higher heterogeneity.

(6) $$RMSD=\sqrt{\frac{1}{n}\sum {\left({\vec{z}}_{1i}-{\vec{z}}_{2i}\right)}^{2}}$$

**Figure 12:**
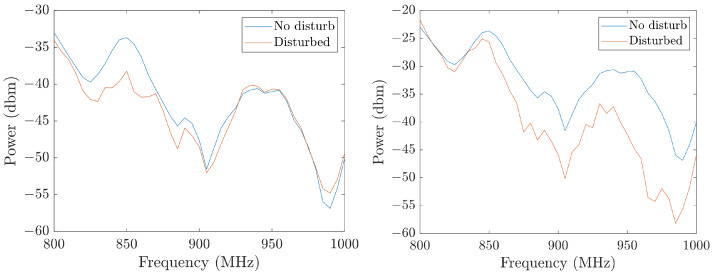
The variation caused by an external electromagnetic interference at x=0 on the one-loop transmitter (**left**) and the multi-loop transmitter (**right**).

Second, we tested the robustness of the two designs with additional interference. While the transmitter and receiver are fixed, we intentionally swung the cable connected to the transmitter to create this interference whose amplitude could reach 20% of the reference signal. Then we calculated the RMSD between the disturbed measurement as ${\vec{z}}_{1}$ and the undisturbed measurement as ${\vec{z}}_{2}$. The RMSDs at the five locations were then averaged as before. Figure 12 shows the impact of the disturbance at location x=0 as an example. The averaged RMSDs among all locations are 1.74 and 6.80 for the one-loop antenna and the multi-loop antenna, respectively. These two experiments illustrate that the antenna with higher electrical length has higher heterogeneity but lower robustness. In practice, the different antenna shapes fit well in different situations. For example, the one-loop antenna is better if the environment is highly noisy, while the multi-loop antenna is better if higher locating resolution is needed. In the rest of this paper, we chose the one-loop antenna transmitter as an illustration. The antenna design can be refined in the future to achieve a better balance between these two factors.

**Figure 13:**
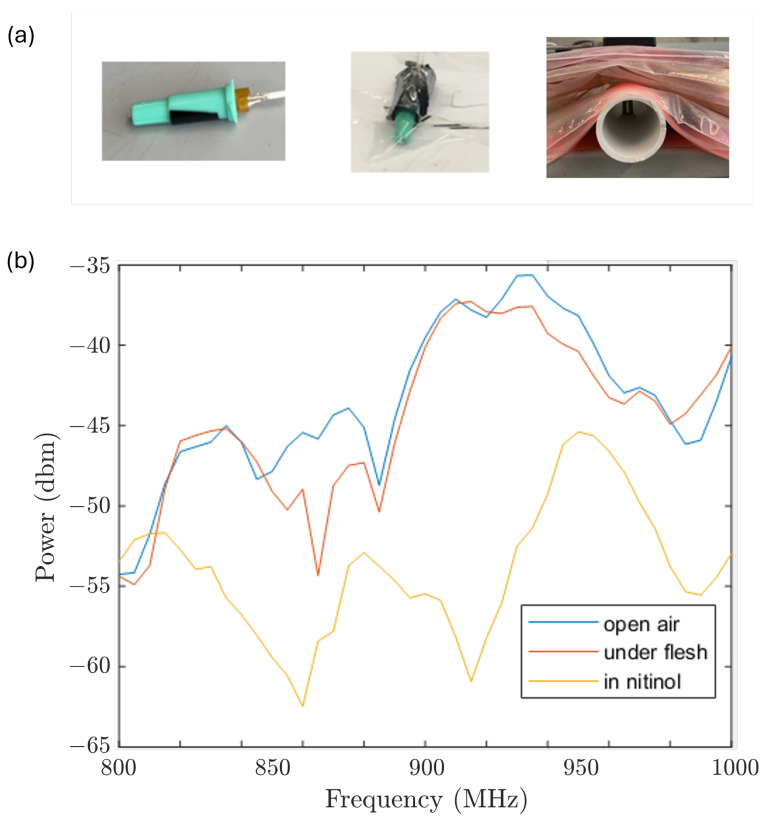
The effect of objects between the transmitter and the receiver. (**a**) Left: Only plastic catheter. Middle: The plastic catheter covered by Nitinol foil. Right: The plastic catheter covered by artificial flesh. (**b**) Measurements from these conditions. The signal was distorted by the Nitinol foil.

All previous simulations were assumed to be under the vacuum condition with no objects obstructing the field between the transmitter and the receiver. Next, we considered the specific absorption rate (SAR) in practice, which is mainly generated by the human body as an obstruction between the receiver and the transmitter. Another disturbing object is the stent, which is made of Nitinol (nickel titanium), a bio-compatible but EMF reflective material. Instead of measuring the SAR directly, we tested their impact on the measured signal strength under three conditions shown in Figure 13a, where the receiver is housed in a plastic catheter, a catheter covered with a Nitinol foil, and a catheter covered with layers of artificial flesh. The results of Figure 13b suggest that the flesh layers do not cause a significant difference, while the Nitinol foil does. To prevent the stent from causing distortion in the reference EMF, the receiver was separated axially from the Nitinol stent along the guide wire by 2 cm, which is long enough to exclude the impact of distortion.

### 4.2. Location Obtained with Deep Learning

**Figure 14:**
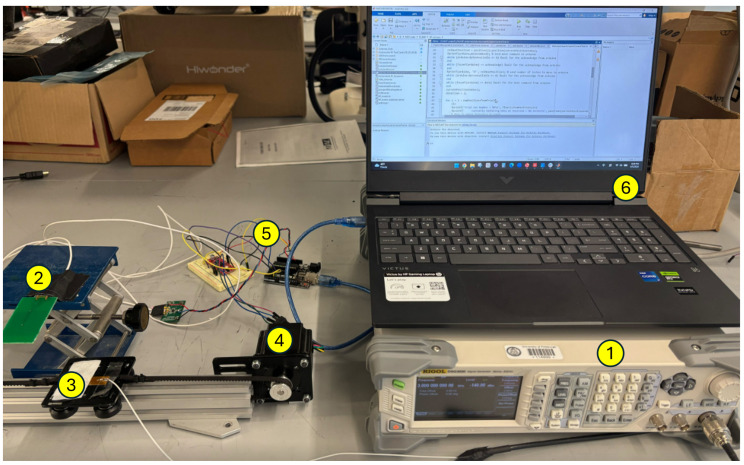
The automated data collection system. The devices are: 1. Function generator 2. Transmitter 3. Receiver 4. Stepper motor 5. Arduino 6. Computer.

**Figure 15:**
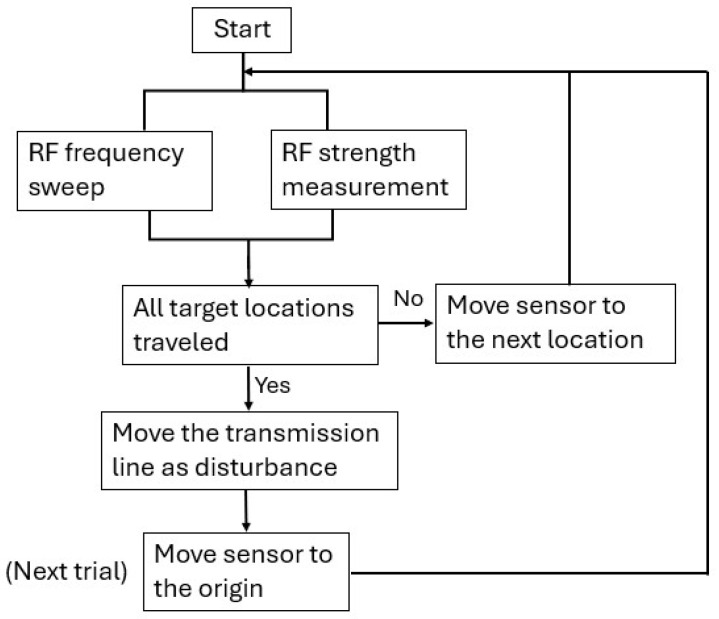
The automated data collection workflow of repeated trials with varying disturbance for the DL model training.

**Figure 16:**
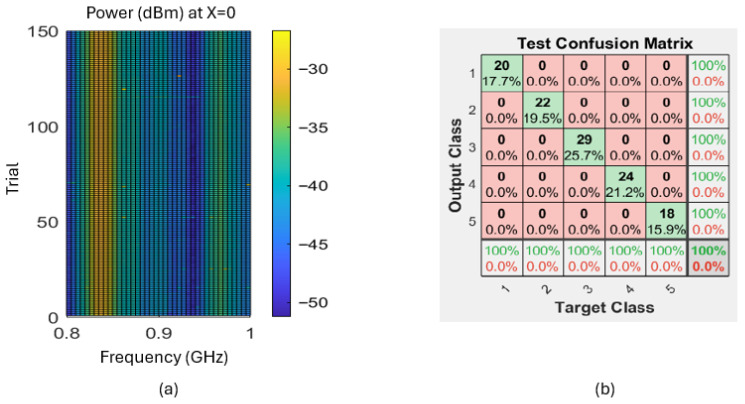
(**a**) Measurements from 150 trials at X=0 shows restricted interference. (**b**) Prediction confusion matrix for the test group by a random split.

All devices used in this system were placed in the apparatus as shown in Figure 14. The transmitter is placed at a fixed location, while the receiver is moved on a straight rail driven by a stepper motor to reference locations. An Arduino controls the stepper motor and reads the power measurement from the receiver. The computer controls the function generator to create the desired frequency spectrum. One limitation of this apparatus is that the orientation of the sensor cannot be adjusted freely. Therefore, we excluded the potential impact of rotation change during the sensor’s movement. While in practice, the stepper motor is not used. Once the transmitter is set up at the same height as it is in the lab condition, measurements can be obtained immediately as the RF frequency sweep starts. With this apparatus, the training data collection workflow is shown in Figure 15. In each trial, the stepper motor moves the receiver antenna to *x* locations of −5,−2.5,0,+2.5,+5 cm, and at each location, the transmitter frequency *f* is swept from 800 MHz to 1000 MHz with increments of 5 MHz. This procedure generates 41 features at each target location. With this automated system, we performed the above procedure for a total of 150 trials to obtain data with which to train the DL network. The receiver wire was not restricted during the repeated motion, which was the main cause of random noise. The data collection process took place throughout the day, with other devices such as smartphones and computers around, which added to the random noise. In addition, as another source of disturbance, the random movement of the transmission line could follow a certain non-Gaussian distribution, which does not perfectly satisfy the independent noise assumption. For the one-loop antenna whose averaged spectrum is shown in Figure 11, the measurements from all trials at location X=0 are shown in Figure 16a. 70% of the trials were used as the training set, 15% as the validation set, and 15% as the test set. When the raw measurements were used as the input to the DL model introduced in Section 3.4, the classification reached 100% accuracy in prediction, as shown in Figure 16b, which illustrates the feasibility of the locating algorithm and the system’s robustness against most common sources of interference.

**Figure 17:**
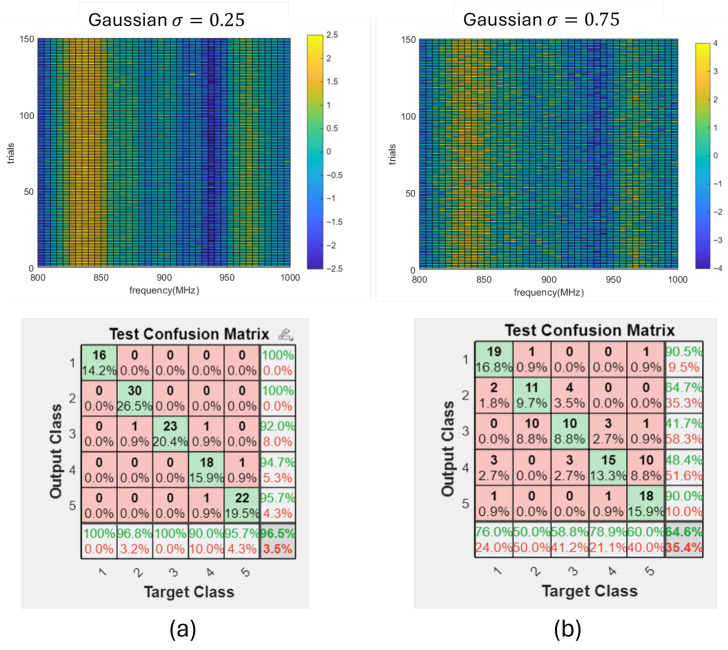
The measurements in Figure 16 with additional Gaussian noise and prediction confusion matrix on the test group. (**a**) σ=0.25. (**b**) σ=0.75.

Next, we tested the system robustness by introducing amplified noises in different forms for *W* in Equation (4). First, we assumed that the noise follows a zero-mean Gaussian distribution $N(0,\sigma^{2})$. Noise is added to the normalized input features (with its standard deviation σ=1). By adding noise with different values of σ, we could learn the robustness of the system with respect to the signal-to-noise ratio. With the contaminated signal used in both training and testing, the change in prediction precision is presented in Table 2, of which two examples with σ=0.25 and σ=0.75 are shown in Figure 17. Statistically, when σ<0.25 (or SNR >4), we can say that the measurements are still reliable, since the accuracy is still higher than the 95% confidence level.

**Figure 18:**
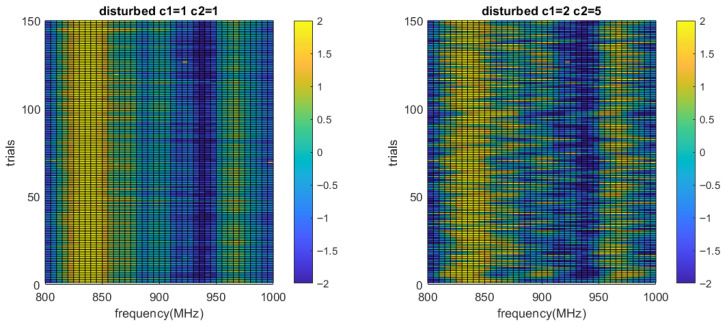
The measurements in Figure 16 with additional sinusoidal noise with different amplitude and frequency.

Another form of *W* was assumed to be sinusoidal, as expressed by Equation (7). This form represents disturbances that vary in a continuous, repetitive pattern, such as reflective surgical tools moving back and forth around the sensor or the swings of the transmission line in a moving vehicle. In this model, the SNR is determined by the value of $C_{1}$, but the overall impact of the noise is also relative to $C_{2}$ and $C_{3}$. Among all the trials, we added the noise signal with a random number for $C_{1}$, $C_{2}$ and $C_{3}$ sampled from uniform distributions ranging from 1 to $c_{1}$, $c_{2}$ and $c_{3}$, respectively. The disturbed measurements for all trials are shown in Figure 18.

(7) $$W=C_{1}\sin\left(C_{2}2\pi f+C_{3}\right)$$

We noticed that for any pairs of $C_{1}$ and $C_{2}$, if they are introduced into both training and testing data sets, the prediction accuracy remains at a very high level with little variation. For example, when we set $c_{1}=1$ and $c_{2}=1$, the prediction is correct for 111 samples and wrong for 1 sample. When $c_{1}=2$ and $c_{2}=5$, the incorrect prediction number increases only by 1 even if the SNR is significantly reduced. However, the actual noise in practice cannot be perfectly modeled during training. Therefore, we also simulated when the testing set has an unseen pattern in the training set. We generated 5 subsets where $c_{1}=1$, $c_{3}=0$, and $c_{2}$ were integers ranging from 1 to 5. In a five-fold cross test, four subsets are used as training, and the remaining subset is used for testing. For each subset used as the test group, the prediction accuracies are shown in Table 3. This experiment shows that the prediction accuracy drops if the noise pattern is completely unseen during training. It also shows that when the unseen noise amplitude lies within the bounds of the training data ($C_{2}=2,3,4$), the prediction will have higher accuracy than in the situation when the noise amplitude lies outside the training data bounds ($C_{2}=1,5$).

## 5. Conclusions and Limitations

The goal of this research was to develop a portable and reliable locating system for tracking a stent within the human body for use in emergency situations. This research focused on the use of radio frequency signals for measurement, specifically on the circuit and antenna design to generate high-resolution, robust EMF measurements, which were further shown to be feasible for location measurement using a neural network. At this point, the developed system is a successful proof of concept that can measure the location in one dimension at a resolution of 2.5 cm with 100% accuracy under benchtop conditions with common levels of noise. In the presence of amplified Gaussian noise, the system maintains prediction accuracy at 96.5% when the SNR is greater than 4.

There are several limitations to this study. First, the system was used only to measure location in one direction because we measured only the signal amplitude. Other information such as location in the *Y* and *Z* directions and stent orientation can be of interest in other applications and can be obtained if multiple misaligned antenna loops are included in either the receiver or the transmitter. The gradient of the spatial RF strength could be used to address additional location or rotation dimensions. In addition, the localization resolution in the proof-of-concept experiments is not high enough for clinical practice, where the desired resolution is less than 1 cm. Second, the DL denoising algorithm requires noise to be statistically independent across all measurements, which may not hold in practice. For future work, applying more advanced, universally feasible algorithms is a promising direction for improving the system to meet practical requirements.

## Figures and Tables

**Table 2:** The change in prediction accuracy with increased Gaussian noise amplitude.

σ	0	0.25	0.5	0.75	1.0
Precision (%)	100	96.5	77.9	64.6	58.4

**Table 3:** The change of prediction accuracy with increased sinusoidal noise frequency.

The unseen subset ($C_{2}$ value)	1	2	3	4	5
Accuracy (%)	71.3	96.5	89.2	88.0	76.9

## Data Availability

The code and data in this paper can be found at https://github.com/YifanZ94/RF_stent_tracker (accessed on 5 March 2026).
